# Clinical diagnostic value of serum CEA and CA19–9 in patients with advanced pancreatic cancer: A prognostic cohort study

**DOI:** 10.3389/fonc.2026.1893201

**Published:** 2026-09-25

**Authors:** Jianjun Song, Fuping Kang, Lan Ma, Aidi Zhang, Fangfang Yang, Qing Liu

**Affiliations:** 1 Department of Hepatobiliary Surgery, General Hospital of Ningxia Medical University, Yinchuan, China; 2 General Surgery Department, The First People’s Hospital of Shizuishan City, Shizuishan, China

**Keywords:** advanced pancreatic cancer, Carbohydrate antigen 19−9 (CA19−9), Carcinoembryonic antigen (CEA), clinical diagnosis, diagnostic biomarker, prognostic efficacy

## Abstract

**Objective:**

To evaluate the clinical diagnostic value of serum tumor markers (CEA and CA19‑9) and other blood‑based indicators in patients with advanced pancreatic cancer, and to assess their prognostic significance.

**Methods:**

A retrospective analysis of 136 patients with advanced pancreatic cancer was conducted. Preoperative blood test results and peripheral immune cell counts, clinical characteristics, and survival data were collected. Univariate and multivariate Cox regression analyses were performed, and overall survival (OS) was assessed using the Kaplan–Meier method.

**Results:**

ROC analysis showed good diagnostic accuracy for CA19‑9 (AUC = 0.886; optimal cutoff 37.44 U/mL) and CEA (AUC = 0.858; optimal cutoff 7.16 ng/mL). During follow‑up, 98 deaths occurred. Univariate analysis identified lymph node metastasis, elevated LDH, ALP, CA19‑9, CEA, CA125, CRP, NLR, and decreased LMR and albumin as significant risk factors for OS. Multivariate analysis revealed that elevated CA19‑9 (HR = 2.059, 95% CI: 1.185–3.576, *P* = 0.010) was an independent risk factor for survival, whereas CEA was not (HR = 1.309, *P* = 0.266). No significant differences were observed between CA19‑9 groups in tumor stage or metastasis (all *P* > 0.05), except for a borderline difference in tumor size (*P* = 0.078). For CEA groups, no clinicopathological variable differed significantly (all *P* > 0.05). Kaplan–Meier analysis showed significantly lower OS in patients with CA19‑9 ≥ 37.44 U/mL or CEA ≥ 7.16 ng/mL (log‑rank *P* = 0.0004 and *P* = 0.006, respectively).

**Conclusions:**

Preoperative serum CA19‑9 is an independent prognostic factor and a valuable diagnostic biomarker in advanced pancreatic cancer. CEA shows diagnostic potential but is not an independent prognostic factor after adjusting for CA19‑9.

## 1 Introduction

Pancreatic cancer, a highly malignant tumor originating from the gastrointestinal tract, ranks seventh in mortality among common malignant tumors. As of 2024, the five-year survival rate is a mere 13%, positioning it at the bottom among all solid tumors (1, 2). In recent years, the incidence of pancreatic cancer has increased, with projections indicating that it will become the second most common cause of cancer-related deaths by 2030 (3). Despite medical advancements extending the survival of many cancer patients over the past decade, those with advanced pancreatic cancer have seen little benefit. Defined by major blood vessel invasion (T4) or distant metastasis (M1) to organs such as the liver, lungs, and bones (4), advanced pancreatic cancer is often diagnosed late due to challenges in screening (5). Moreover, resistance to radiotherapy, targeted therapies, and immunotherapies exacerbates the prognosis. Currently, the median survival for pancreatic cancer patients is under one year, with a five-year survival rate of approximately 8% overall, and only 3% for those with distant metastases (6).

Early diagnosis of advanced pancreatic cancer remains a significant clinical challenge, owing to its insidious onset and nonspecific symptoms. While imaging and pathological biopsy are the gold standards for diagnosis, their application typically occurs only after patients exhibit symptoms. Serum biomarkers, such as CEA and CA19-9, could support earlier clinical diagnosis and risk stratification; however, the diagnostic value of these markers in advanced pancreatic cancer has not been fully established. This study, therefore, aims to investigate the clinical diagnostic utility of CEA and CA19-9, along with other blood indicators, in patients suffering from advanced pancreatic cancer.

Treatment strategies for pancreatic cancer seek to eliminate cancer cells while preserving healthy tissue, often employing a combination of approaches. For advanced cases, common treatments include chemotherapy, immunotherapy, targeted therapies, and surgery (7, 8). Gemcitabine, the first approved chemotherapy drug, inhibits cancer cell growth and promotes immune-mediated killing, administered intravenously in cycles until disease progression (9, 10). Immunotherapy utilizing PD-1/PD-L1 inhibitors shows promise but is constrained by the immunosuppressive tumor microenvironment characteristic of pancreatic cancer (11). Targeted therapies, which focus on pathways such as PI3K/AKT and RAS, aim for precise treatment; however, response rates among advanced patients remain low, necessitating the ongoing development of new therapies (12).

Tumor markers, specific molecules produced by tumor cells and detectable in a patient’s body fluids or tissues, serve as crucial references for the clinical diagnosis and treatment of malignant tumors (13, 14). Serum tumor marker detection, a simple and non-invasive method, has become a common clinical screening approach. These markers are invaluable for early tumor diagnosis, differential diagnosis, and prognosis assessment (15). However, pancreatic cancer, characterized by heterogeneity and involving multiple genetic and epigenetic changes, lacks organ specificity and often results in high false-positive rates. This leads to underdiagnosis and misdiagnosis, significantly impacting medical diagnosis and treatment (16). Currently, no single blood-based biomarker possesses sufficient sensitivity or specificity for routine pancreatic cancer screening, and no reliable sole diagnostic criterion exists (17). Thus, the search for serum-specific tumor markers is of utmost importance.

In recent years, with rapid advancements in molecular biology, tumor markers such as alpha-fetoprotein (AFP), carcinoembryonic antigen (CEA), carbohydrate antigen 72-4 (CA72-4), and carbohydrate antigen 19-9 (CA19-9) have been widely applied in diagnosing various cancers, including primary hepatocellular carcinoma, colorectal cancer, and pancreatic cancer (18–21). Notably, CA19–9 has received FDA approval for diagnosing pancreatic cancer. Clinically, CEA, along with metrics such as glucose and amylase, is also utilized in conjunction with CA19-9. CA19-9 is particularly valuable for the early detection of pancreatic cancer and monitoring disease progression, while CEA, although less specific for pancreatic cancer when assessed alone, can be combined with other markers for a more comprehensive evaluation. Glucose levels may be abnormal in some pancreatic cancer patients due to disrupted pancreatic function affecting insulin production. Additionally, amylase, produced by the pancreas, can provide insights into pancreatic health, with elevated levels sometimes associated with pancreatic disorders, including cancer. Nevertheless, the overall sensitivity of CA72-4, CA19-9, and CEA, whether individually or in combination, remains insufficient for the screening and identification of pancreatic cancer (22, 23). Therefore, exploring non-invasive and highly specific serum biomarkers for early pancreatic cancer detection and mortality reduction holds significant clinical value.

This study aims to explore the prognostic value of blood test indices for patients with advanced pancreatic cancer and establish a survival analysis to provide more comprehensive treatment recommendations and management plans.

## 2 General information and methods

### 2.1 General information

Retrospectively, 136 patients with advanced pancreatic cancer in the Department of Hepatobiliary Surgery of General Hospital of Ningxia Medical University from July 2020 to December 2023 (statistical cut-off date was December 31, 2023) were analyzed, and the specific clinical data are shown in **Table 1**, of which 97 (71.3%) were male and 39 (28.7%) were female. The median follow-up time was 6.84 months (1.75-11.96 months) and 98 patients died before the final follow-up date. The mean age was 65.84 ± 7.43 years (range 24.56 ~ 79.31). The mean body mass index (BMI) was (25.62 ± 6.89). A total of 112 patients with advanced pancreatic cancer received postoperative adjuvant radiotherapy; 89 chemotherapeutic regimens were predominantly used for patients with stage III pancreatic cancer, and 47 chemotherapeutic regimens were predominantly used for patients with stage IV pancreatic cancer. The study was approved by the Ethics Committee of General Hospital of Ningxia Medical University, and all study participants signed an informed consent form.

**Table 1:** Baseline clinical characteristics of patients with advanced pancreatic cancer (n = 136).

Variable	Statistic
Age (years)	66.46 ± 7.11
BMI (kg/m²)	26.76 ± 6.24
Tumor size (mm)	43.11 ± 8.79
CEA (ng/mL)	7.25 ± 2.54
CA19-9 (U/L)	39.96 ± 12.04
LDH (U/L)	485.96 ± 102.98
ALP (U/L)	309.24 ± 73.80
CA125 (U/mL)	9.68 ± 2.21
CA242 (U/mL)	3.11 ± 1.50
CA724 (U/mL)	1.57 ± 0.17
CRP (mg/L)	28.12 ± 5.83
D-dimer (μg/L)	895.32 ± 100.06
Albumin (g/L)	18.07 ± 5.69
Neutrophil (×10^9^/L)	4.58 ± 0.96
Lymphocyte (×10^9^/L)	1.95 ± 0.30
Platelet (×10^9^/L)	280.45 ± 33.54
Monocyte (×10^9^/L)	0.38 ± 0.11
NLR	2.41 ± 0.68
LMR	5.74 ± 2.36
PLR	147.24 ± 29.41
Gender (Male)	93 (68.4%)
Tumor stage (IV)	50 (36.8%)
Local lymph node metastasis (Yes)	112 (82.4%)
Distant metastasis (Yes)	96 (70.6%)
Postoperative chemotherapy (Yes)	106 (77.9%)
Gemcitabine protocol (Yes)	102 (75.0%)

Data are presented as mean ± standard deviation or n (%).

BMI, Body Mass Index; CEA, Carcinoembryonic antigen; LDH, Lactate dehydrogenase; ALP, Alkaline phosphatase; CA19-9, Carbohydrate antigen 19-9; CA125, Carbohydrate antigen 125; CA242, Carbohydrate antigen 242; CA724, Carbohydrate antigen 724; CRP, C-reactive protein; ALB, albumin.

### 2.2 Inclusion and exclusion criteria

Inclusion criteria: ① Pancreatic primary foci or metastatic foci have been subjected to puncture biopsy and diagnosed as pancreatic ductal adenocarcinoma by pathological histological examination; ② According to the TNM staging criteria of pancreatic cancer of the 7th edition of the AJCC/UICC in 2010, the diagnosis is Stage III (**Figure 1A**) or Stage IV (**Figure 1B**) (i.e., locally advanced or metastatic cancer); ③ General condition is good, with the score of ECOG being 0-1, and no serious heart, lung, brain, liver, kidney and other important organ diseases or functional defects; ④ No chemotherapy, radiotherapy, interventional therapy, immunotherapy or other related anti-tumour treatments after diagnosis of pathological diagnosis; For patients receiving adjuvant therapy (112/136 patients), a clearly documented adjuvant therapy status that does not interfere with the assessment of advanced first-line chemotherapy observed in this study is required; ⑤ Good general condition, no serious heart, lung, brain, liver, kidney or other important organ diseases or functional defects, and able to receive at least two cycles of advanced first-line chemotherapy; ⑥ Complete clinical and follow-up data, including gender, age, TNM stage of the tumour, treatment plan, laboratory test indexes, imaging data, and overall survival time, etc., and the chest CT and abdominal imaging are taken before the first anti-tumour treatment. Up to the date of the last follow-up visit, there are no tumours of other parts of the body of the patient which are concurrent or heterogeneous.

**Figure 1:**
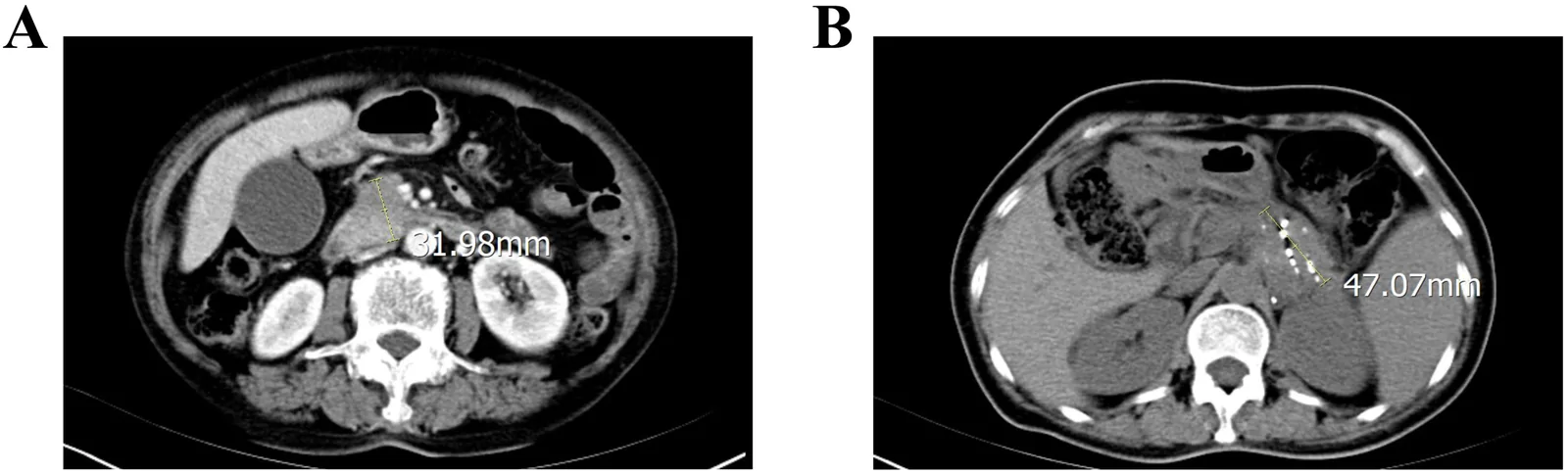
Representative computed tomography (CT) images of patients with advanced pancreatic cancer. **(A)** Stage III disease (locally advanced, no distant metastasis); **(B)** Stage IV disease (with distant metastasis).

Exclusion criteria: ① patients with other tumours; ② patients with abnormal liver and kidney functions and blood diseases; ③ patients who have received chemotherapy and radiotherapy in the past; ④ patients with bleeding tendency and psychiatric disorders; ⑤ patients who lost their visits during the follow-up period. All enrolled patients had pathologically confirmed PDAC; patients with other pancreatic lesions, including IPMN, were excluded.

### 2.3 Laboratory tests and related clinical indicators

Fasting venous blood samples were collected from the patients’ elbows by a nurse within two weeks prior to surgery and sent to the Laboratory Department for analysis of peripheral blood and complete blood counts.

Laboratory tests included serum lactate dehydrogenase (LDH), alkaline phosphatase (ALP), carbohydrate antigens 724 (CA724), 242 (CA242), and 125 (CA125), CEA, CA19-9, C-reactive protein (CRP), D-dimer, and albumin (ALB). The critical values for serum LDH, ALP, CA19-9, CA125, CA242, CA72-4, CRP, and D-dimer were defined as the upper limit of normal (ULN), with levels exceeding the ULN indicating elevation. The critical values for serum ALB were defined as the lower limit of normal (LLN), where levels below the LLN indicated decreased levels. All serum biomarkers were measured using commercially available electrochemiluminescence immunoassay kits on a Cobas e601 analyser (Roche Diagnostics, Mannheim, Germany), following the manufacturer’s protocols. Blood samples, collected in serum separation tubes, were centrifuged at 3,000 rpm for 10 minutes within two hours of collection, and aliquoted serum was stored at −80 °C until batch analysis. All assays were conducted by trained laboratory technicians who were blinded to patient clinical data, with intra-assay and inter-assay coefficients of variation remaining below 5% and 8%, respectively, for all markers.

### 2.4 Surgical criteria and types of surgery

Inclusion of patients with advanced pancreatic cancer undergoing surgery was based on strict criteria: (1) tumour resectability assessment. A multidisciplinary team (MDT) comprising pancreatic surgeons, imaging specialists, and oncologists conducted comprehensive resectability assessments according to internationally accepted standards, such as the National Comprehensive Cancer Network (NCCN) guidelines. Surgical resection was considered feasible for patients whose tumours did not invade major blood vessels, such as the celiac trunk and superior mesenteric artery, and who exhibited limited distant metastases (i.e., isolated liver metastases with ≤ 3 foci or confined to regional lymph nodes). (2) Physical condition. Patients must have been in good physical condition, with an Eastern Cooperative Oncology Group (ECOG) performance score of ≤ 2, and essential organ functions (e.g., heart, lungs, liver, kidneys) capable of tolerating surgical trauma.

Surgical types included: (1) Whipple resection: This was the primary surgical option for patients with pancreatic head cancer meeting the specified criteria, with 67.7% (92/136) of patients undergoing this procedure. The surgical protocol adhered to standardized operating procedures to ensure complete resection of the tumour and surrounding tissues, alongside careful gastrointestinal tract reconstruction. (2) Pancreatic tail resection: This procedure was performed for patients with tumours confined to the tail of the pancreas and without extensive distant metastasis, representing 22.8% (31/136) of surgeries. Intraoperative focus was placed on the clearance of splenic vessels and surrounding lymph nodes. (3) Palliative surgery: For patients unable to achieve radical resection but experiencing biliary or digestive tract obstruction symptoms, palliative surgery was conducted to alleviate symptoms and enhance quality of life. This included biliary and intestinal anastomosis, along with gastrointestinal anastomosis, and accounted for 9.6% (13/136) of cases.

### 2.5 Patient follow-up

All patients were followed up via telephone or during hospitalization, with follow-up concluding in October 2023. The study endpoint was defined as the time to patient death. Overall survival (OS) represented the duration from the date of surgery to death or the end of follow-up. Instances of patient refusal to participate in follow-up, withdrawal for various reasons (e.g., involvement in another clinical study, occurrence of unrelated death, etc.), and loss of contact were classified as loss to follow-up.

### 2.6 Statistical analyses

Data analysis was conducted using IBM SPSS version 26.0 for Mac and R version 3.6.3 (R Project). A *P*-value of < 0.05 indicated a significant difference between groups. Categorical variables were compared using the chi-square test or Fisher’s test. The optimal cut-off values for CA19–9 and CEA were determined using the maximum Youden index derived from the receiver operating characteristic (ROC) curve, with the area under the curve (AUC) calculated accordingly. Univariate and multivariate analyses were performed using Cox regression models. Survival analyses utilized Kaplan-Meier analysis, with between-group comparisons assessed using the log-rank (LogRank) test. ROC curves were employed to evaluate the diagnostic accuracy of CA19–9 and CEA in identifying high-risk patients with advanced pancreatic cancer.

## 3 Results

### 3.1 Univariate Cox proportional risk regression modelling to analyse risk factors affecting the prognosis of patients with advanced pancreatic cancer

In this study, a Cox proportional hazards regression model was employed to conduct a univariate analysis of various clinicopathological parameters, including age, gender, tumour stage, chemotherapy regimen, and serum tumour metabolism indices, along with tumour markers, systemic inflammation indices, coagulation indices, and nutritional status indices of the patients prior to anti-tumour therapy. The analysis results indicated that several factors were associated with OS in patients with advanced pancreatic cancer, including localized lymph node metastasis (*P* = 0.001), elevated serum LDH levels (*P* < 0.001), elevated ALP levels (*P* < 0.001), elevated CA19–9 levels (*P* = 0.001), elevated CEA levels (*P* = 0.001), elevated CA125 levels (*P* = 0.015), elevated CRP levels (*P* < 0.001), elevated neutrophil-to-lymphocyte ratio (NLR) (*P* = 0.001), lowered lymphocyte-to-monocyte ratio (LMR) (*P* = 0.028), and decreased albumin (ALB) levels (*P* = 0.010), as detailed in **Table 2**.

**Table 2:** Univariate Cox regression analysis for overall survival.

Variable	HR (95% CI)	P value
Age	0.985 (0.958–1.012)	0.271
BMI	0.998 (0.967–1.031)	0.918
Tumor size	1.005 (0.983–1.028)	0.641
CEA (ng/mL)	2.672 (1.528–4.706)	0.001
CA19-9 (U/L)	2.079 (1.374–3.416)	0.001
LDH	4.847 (2.846–8.284)	< 0.001
ALP	2.973 (1.925–4.865)	< 0.001
CA125	1.436 (0.895–2.574)	0.015
CA242	1.009 (0.883–1.154)	0.892
CA724	2.024 (0.565–7.247)	0.279
CRP	3.328 (2.053–5.337)	< 0.001
D-dimer	1.001 (0.999–1.003)	0.234
Albumin	1.725 (1.227–2.308)	0.010
Neutrophil	0.803 (0.650–0.992)	0.042
Lymphocyte	1.043 (0.563–1.933)	0.893
Platelet	0.994 (0.988–1.001)	0.080
Monocyte	0.697 (0.121–4.012)	0.686
NLR	2.236 (1.387–3.593))	0.001
LMR	1.597 (1.035–2.442)	0.028
PLR	0.995 (0.989–1.002)	0.164
Gender (Male vs Female)	1.192 (0.782–1.817)	0.415
Tumor stage (IV vs III)	1.154 (0.766–1.738)	0.494
Local lymph node metastasis (Yes vs No)	3.295 (1.547–6.598)	0.001
Distant metastasis (Yes vs No)	0.836 (0.546–1.280)	0.410
Postoperative chemotherapy (Yes vs No)	1.279 (0.771–2.122)	0.340
Gemcitabine protocol (Yes vs No)	0.836 (0.525–1.332)	0.451

HR, hazard ratio; CI, confidence interval.

### 3.2 Multivariate Cox regression analysis of risk factors affecting overall survival in patients with advanced pancreatic cancer

The multivariate Cox regression analysis included all variables with *P* < 0.1 from the univariate analysis. It revealed that an elevated CA19–9 level (≥ 37.44 U/mL) was an independent risk factor for OS, showing a hazard ratio (HR) of 2.059 (95% CI: 1.185–3.576, *P* = 0.010). Conversely, the elevated CEA level (≥ 7.16 ng/mL) did not reach statistical significance in the multivariate model, with an HR of 1.309 (95% CI: 0.815–2.104, *P* = 0.266), as shown in **Table 3**.

**Table 3:** Multivariate Cox regression analysis for overall survival.

Variable	HR (95% CI)	P value
CA19-9 (≥37.44 U/mL)	2.059 (1.185–3.576)	0.010
CEA (≥7.16 ng/mL)	1.309 (0.815–2.104)	0.266

Variables selected for multivariate analysis: CA19-9 and CEA based on their optimal cut-off values obtained from ROC analysis.

### 3.3 Relationship between serum CA19–9 levels and clinicopathological parameters

Using the optimal cut-off value of 37.44 U/mL for CA19-9, patients were categorized into two groups: low CA19-9 (< 37.44 U/mL, n = 52) and high CA19-9 (≥ 37.44 U/mL, n = 84). As summarized in **Table 4**, no statistically significant differences were observed between the two groups regarding age (*P* = 0.697), local lymph node metastasis (*P* = 1.000), distant metastasis (*P* = 0.758), tumour stage (*P* = 0.889), or chemotherapy protocol (*P* = 0.154). However, a borderline difference was noted in primary tumour size (*P* = 0.078), where a higher proportion of patients in the high CA19–9 group had tumours measuring 4 cm or larger (84.5% vs. 73.1%).

**Table 4:** Comparison of clinicopathological parameters between CA19-9 high and low groups.

Variable	Category	CA19-9 < 37.44 (n = 52)	CA19-9 ≥ 37.44 (n = 84)	Statistic (χ²)	P value
Age	< 60	6 (11.5%)	13 (15.5%)	0.151	0.697
	≥ 60	46 (88.5%)	71 (84.5%)		
Primary focus size	2 cm	0 (0.0%)	2 (2.4%)	5.104	0.078
	3 cm	14 (26.9%)	11 (13.1%)		
	4 cm	38 (73.1%)	71 (84.5%)		
Local lymph node metastasis	No	9 (17.3%)	15 (17.9%)	0.000	1.000
	Yes	43 (82.7%)	69 (82.1%)		
Distant metastasis	No	14 (26.9%)	26 (31.0%)	0.095	0.758
	Yes	38 (73.1%)	58 (69.0%)		
Chemotherapy protocol (gemcitabine)	No	17 (32.7%)	17 (20.2%)	2.034	0.154
	Yes	35 (67.3%)	67 (79.8%)		
Tumor stage	III	32 (61.5%)	54 (64.3%)	0.020	0.889
	IV	20 (38.5%)	30 (35.7%)		

Data are presented as n (%). P values were calculated using the chi-square test (or Fisher’s exact test when appropriate).

CA19-9, Carbohydrate antigen 19-9.

### 3.4 Relationship between serum CEA levels and clinicopathological parameters

Patients were classified based on the optimal cut-off value of 7.16 ng/mL into two groups: low CEA (< 7.16 ng/mL, n = 65) and high CEA (≥ 7.16 ng/mL, n = 71). As shown in **Table 5**, no significant differences were observed in clinicopathological variables between the two groups (all *P* > 0.05), including age (*P* = 0.836), primary tumour size (*P* = 0.397), local lymph node metastasis (*P* = 0.989), distant metastasis (*P* = 0.542), chemotherapy protocol (*P* = 1.000), and tumour stage (*P* = 1.000).

**Table 5:** Comparison of clinicopathological parameters between CEA high and low groups.

Variable	Category	CEA < 7.16 (n = 65)	CEA ≥ 7.16 (n = 71)	Statistic (χ²)	P value
Age	< 60	10 (15.4%)	9 (12.7%)	0.043	0.836
	≥ 60	55 (84.6%)	62 (87.3%)		
Primary focus size	2 cm	1 (1.5%)	1 (1.4%)	1.849	0.397
	3 cm	15 (23.1%)	10 (14.1%)		
	4 cm	49 (75.4%)	60 (84.5%)		
Local lymph node metastasis	No	12 (18.5%)	12 (16.9%)	0.000	0.989
	Yes	53 (81.5%)	59 (83.1%)		
Distant metastasis	No	17 (26.2%)	23 (32.4%)	0.371	0.542
	Yes	48 (73.8%)	48 (67.6%)		
Chemotherapy protocol (gemcitabine)	No	16 (24.6%)	18 (25.4%)	0.000	1.000
	Yes	49 (75.4%)	53 (74.6%)		
Tumor stage	III	41 (63.1%)	45 (63.4%)	0.000	1.000
	IV	24 (36.9%)	26 (36.6%)		

Data are presented as n (%). P values were calculated using the chi-square test (or Fisher’s exact test when appropriate).

CEA, Carcinoembryonic antigen.

### 3.5 Kaplan-Meier survival analysis

Kaplan-Meier survival curves stratified by CA19–9 and CEA levels are presented in **Figures 2** and **3**, respectively. Patients with CA19–9 levels ≥ 37.44 U/mL exhibited a significantly poorer prognosis compared to those with lower levels, with a median OS of 6.7 months versus 7.1 months (log-rank *P* < 0.001). Similarly, patients with CEA levels ≥ 7.16 ng/mL showed worse survival outcomes than those with CEA levels < 7.16 ng/mL, with a median OS of 6.7 months compared to 7.0 months (log-rank *P* = 0.006).

**Figure 2:**
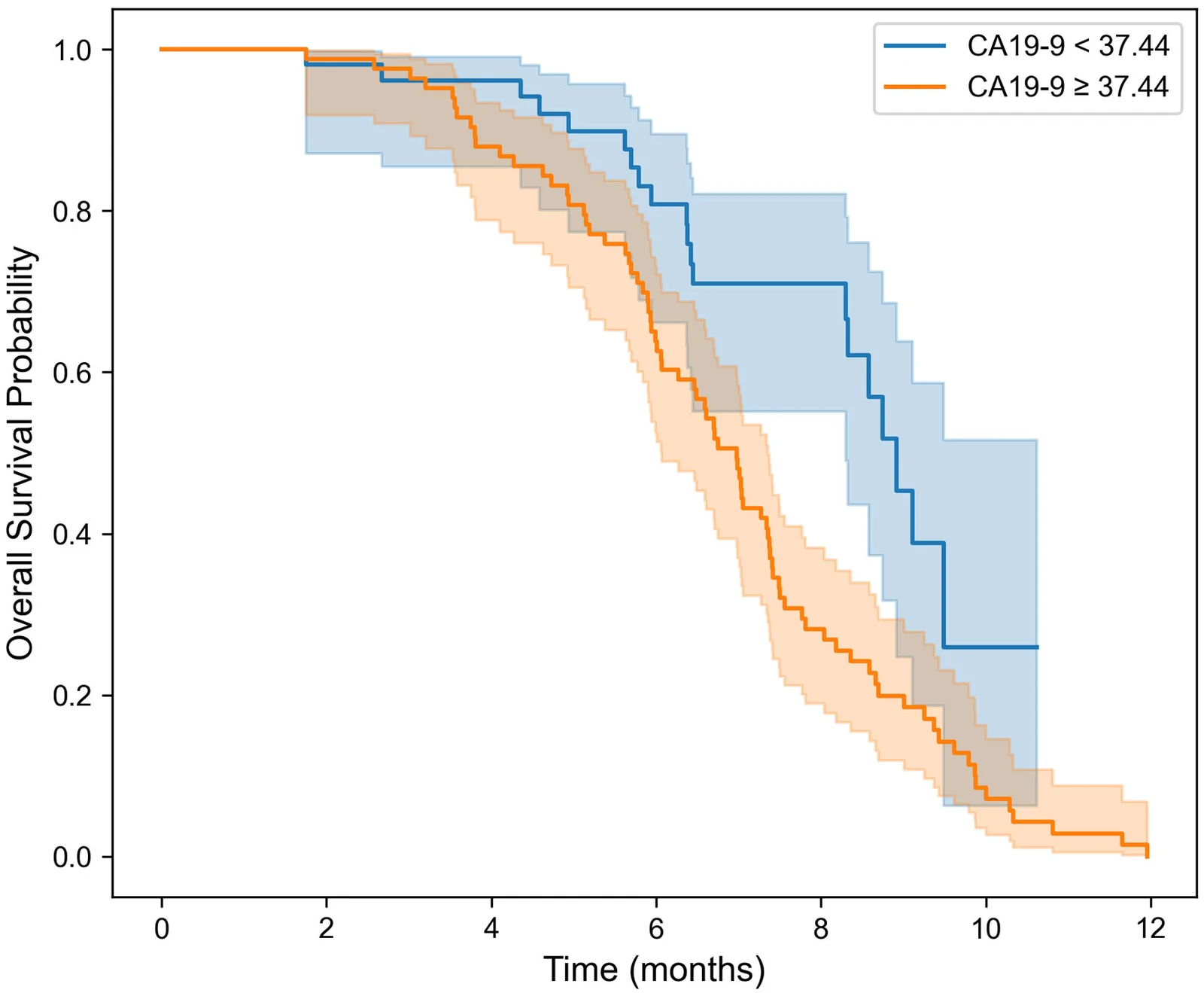
Kaplan–Meier survival curves for overall survival stratified by serum CA19-9 level. Patients were divided into two groups according to the optimal cut-off value of 37.44 U/mL. The high CA19-9 group (≥37.44 U/mL) had a significantly worse prognosis than the low CA19-9 group (log-rank P <0.001). Median overall survival: 6.7 months vs. 7.1 months.

**Figure 3:**
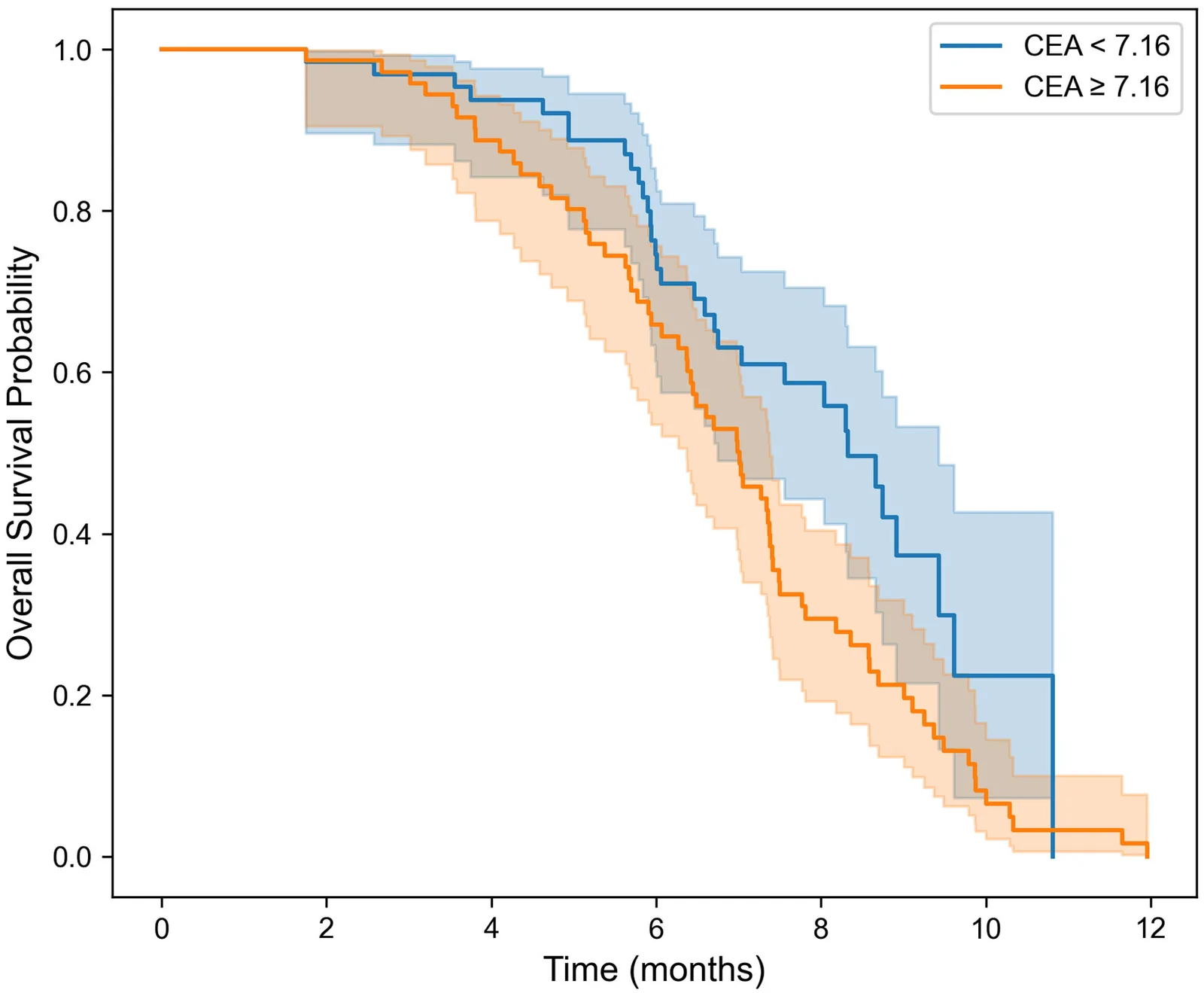
Kaplan–Meier survival curves for overall survival stratified by serum CEA level. Patients were divided according to the optimal cut-off value of 7.16 ng/mL. The high CEA group (≥7.16 ng/mL) showed significantly poorer survival compared with the low CEA group (log-rank P = 0.006). Median overall survival: 6.7 months vs. 7.0 months.

### 3.6 ROC curve analysis of optimal cut-off values for CA19–9 and CEA

ROC curve analysis indicated that CA19–9 provided an AUC of 0.886, with a sensitivity of 79.8% and specificity of 86.5% at the optimal cut-off of 37.44 U/mL. CEA achieved an AUC of 0.858, with a sensitivity of 67.7% and specificity of 89.2% at the optimal cut-off of 7.16 ng/mL (**Figure 4**). The Youden indices for CA19–9 and CEA were 0.663 and 0.569, respectively (**Table 6**).

**Figure 4:**
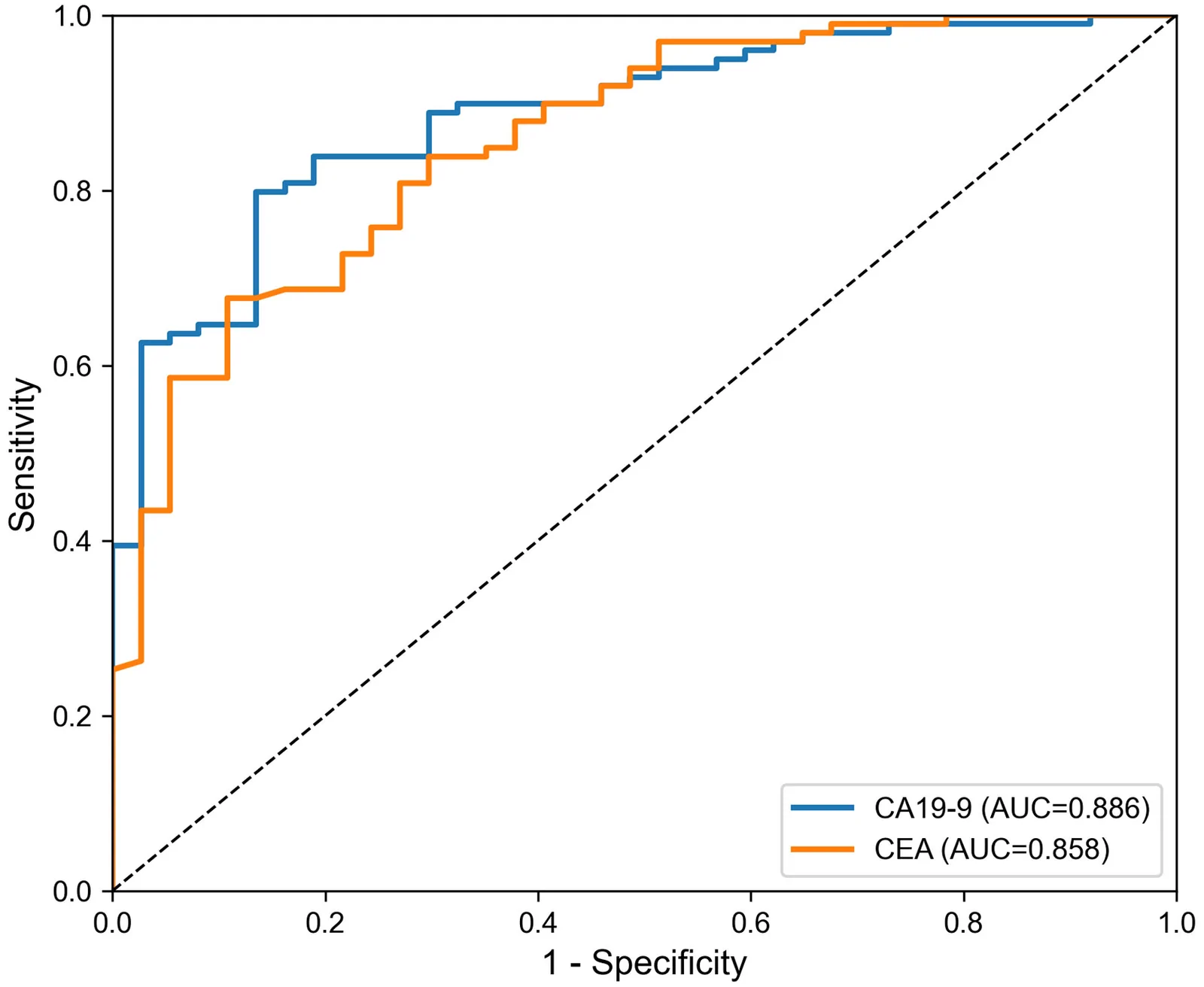
Receiver operating characteristic (ROC) curves of serum CA19-9 and CEA for the diagnosis of advanced pancreatic cancer.

**Table 6:** Receiver operating characteristic (ROC) analysis of CA19-9 and CEA.

Marker	Optimal cutoff	AUC	Sensitivity	Specificity	Youden index
CA19-9	37.44 U/mL	0.886	0.798	0.865	0.663
CEA	7.16 ng/mL	0.858	0.677	0.892	0.569

AUC, area under the curve.

### 3.7 Spearman correlation heatmap

**Figure 5** illustrates a Spearman correlation heatmap of selected serum biomarkers and clinical parameters. The analysis revealed a moderate positive correlation between CEA and CA19-9 (ρ = 0.39). Other inflammatory markers, including CRP, NLR, LMR, and ALB, demonstrated weak correlations with tumour size and with each other (all |ρ| < 0.30). Negative correlations were noted between albumin and several inflammatory indices, yet none achieved statistical significance. Overall, the heatmap illustrates that CEA and CA19–9 are the most closely associated pair among the examined variables, while the remaining parameters display relatively independent patterns.

**Figure 5:**
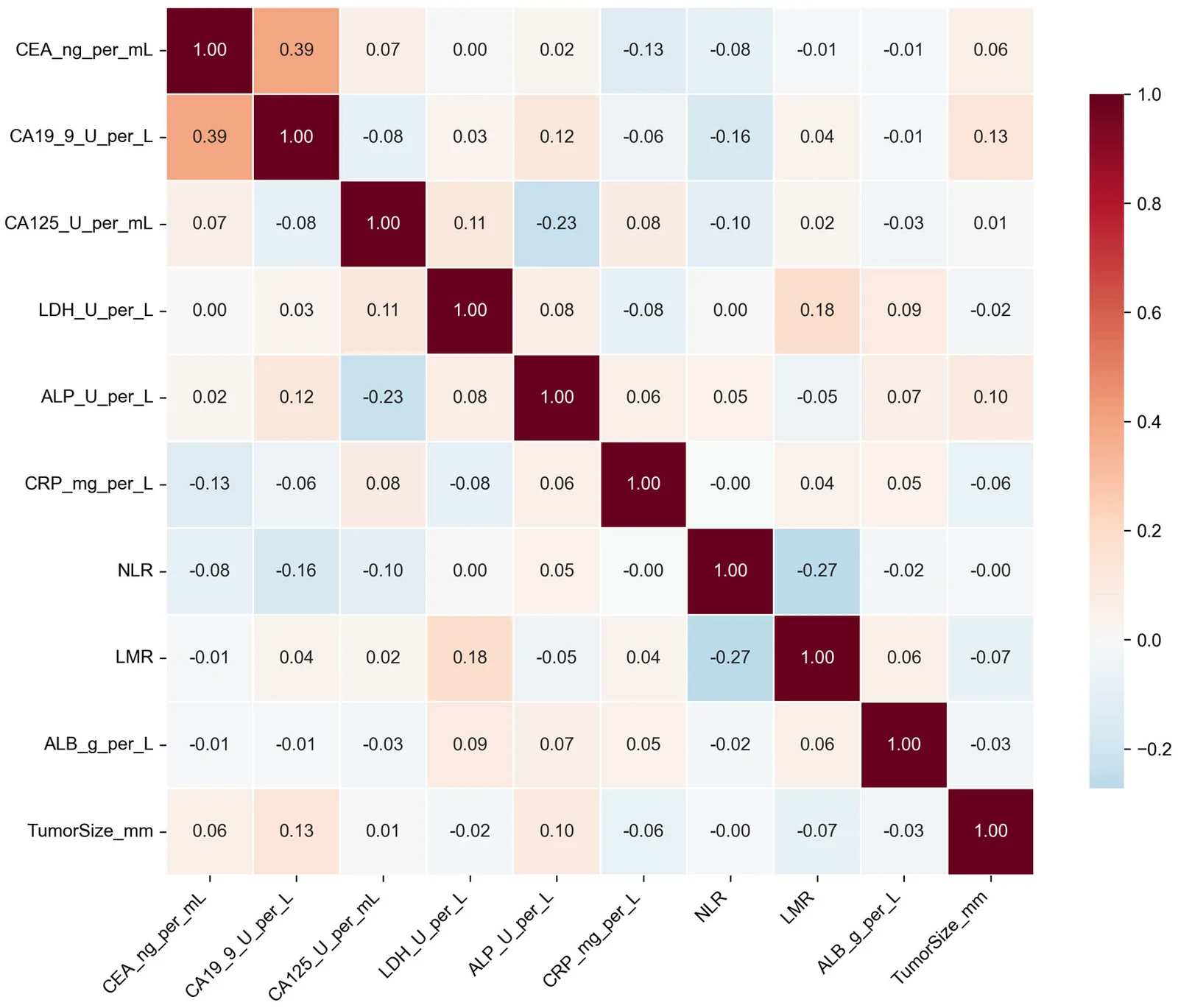
Spearman correlation heatmap of selected serum biomarkers and clinical parameters. The heatmap displays correlation coefficients (ρ) between CEA, CA19-9, CA125, LDH, ALP, CRP, NLR, LMR, albumin, and tumor size. Red indicates positive correlation, blue indicates negative correlation. The strongest positive correlation was observed between CEA and CA19-9 (ρ = 0.39).

## 4 Discussion

Pancreatic cancer, characterized by insidious onset, high malignancy, poor prognosis, and limited responsiveness to conventional radiotherapy and chemotherapy, as well as emerging targeted and immunotherapies, presents a considerable challenge in oncology. Clinical data from the past decade have illustrated a stark lack of substantial progress in improving survival rates for pancreatic cancer patients, with a median survival time of less than one year and a 5-year survival rate under 10% (24). In this context, the exploration of biomarkers for prognostic assessment and the development of survival analysis models for advanced pancreatic cancer patients carry significant academic relevance and are critical for clinical practice. These efforts potentially facilitate personalized patient management, optimize treatment selection, extend survival, enhance quality of life, reduce economic burden, and improve healthcare resource allocation (11). Distinction between conventional serum protein biomarkers, such as CEA and CA19-9, and emerging liquid biopsy markers (e.g., circulating tumour DNA, exosomes, and circulating tumour cells) is crucial. Although liquid biopsy markers show promise for early detection and molecular profiling, their high cost and technical complexity pose challenges for routine clinical applications. In contrast, serum protein markers are widely accessible, cost-effective, and easily integrated into standard clinical workflows, making them more practical for prognosis monitoring in advanced disease settings.

This study meticulously investigates associations between a comprehensive array of indicators and the prognosis of patients with advanced pancreatic cancer. Serum CEA and CA19-9, among the most extensively utilized tumour markers in cancer diagnosis and prognosis prediction (25), serve as central elements in this analysis. Additionally, CA125 has recently been identified as closely related to hepatic metastasis, prognosis, and surgical benefit in pancreatic cancer (26), while CA242 and CA724 are established digestive tract tumour markers (27). Furthermore, serum levels of CRP, ALB, and D-dimer reflect systemic inflammation, nutritional status, and thrombotic risk, respectively (28, 29). Prior research consistently demonstrates the prognostic significance of these laboratory indicators across various malignancies, including pancreatic cancer (30, 31). The inclusion of NLR and LMR, two clinical inflammatory markers, is supported by their well-documented associations with the prognosis of pancreatic cancer patients (32, 33).

The univariate and multivariate Cox proportional hazards regression analyses revealed several critical findings. Univariate analysis identified localized lymph node metastasis, elevated serum LDH, ALP, CA19-9, CEA, CA125, CRP, and NLR, along with decreased LMR and ALB, as significant factors associated with poorer prognosis. Notably, multivariate analysis identified elevated CA19–9 levels as an independent risk factor for survival in patients with advanced pancreatic cancer (HR = 2.059, *P* = 0.010), whereas CEA did not maintain independent significance after adjustment for CA19-9 (*P* = 0.266). This finding reinforces the pivotal roles of CA19–9 and CEA in prognostic assessment and aligns with previous studies in other gastrointestinal cancers. For instance, Zheng et al. (34) reported a strong correlation between CEA and CA19–9 levels and colorectal cancer lesions, where elevated levels of these markers indicated poor prognosis due to lymph node and liver metastases. Additionally, Ermiah et al. (35) demonstrated that CEA and CA19–9 levels could predict death risk in pancreatic cancer patients, effectively guiding the selection of adjuvant therapies. These consistent findings across various gastrointestinal tumours suggest a shared underlying mechanism likely involving the regulation of tumour cell proliferation, invasion, and metastasis. It is hypothesized that high expression levels of CEA and CA19–9 may modulate the tumour microenvironment, facilitating angiogenesis, immune evasion, and, ultimately, disease progression. However, further in-depth basic research is essential to validate this hypothesis.

ROC curve analysis determined optimal cut-off values of 37.44 U/mL for CA19–9 and 7.16 ng/mL for CEA. At these cut-offs, no statistically significant differences were observed in any clinicopathological variables between CA19–9 groups, except for a borderline difference in primary tumour size (*P* = 0.078). For CEA groups, none of the variables demonstrated significant differences (all *P* > 0.05). Kaplan-Meier survival analysis further showed that patients with CA19-9 ≥ 37.44 U/mL or CEA ≥ 7.16 ng/mL had significantly lower OS compared to those with lower marker levels (log-rank *P* < 0.001 and *P* = 0.006, respectively). These findings visually illustrate the strong correlation between CA19–9 and CEA levels and patient survival, offering a practical quantitative tool for clinicians. Clinically, patients with elevated CA19–9 and CEA levels may benefit from more aggressive treatment strategies, such as intensified chemotherapy regimens, novel targeted therapies, or immunotherapy combinations. In contrast, patients with lower marker levels may have treatment intensity adjusted to optimize efficacy while minimizing treatment-related adverse effects and enhancing quality of life.

Notably, the study revealed that preoperative serum CEA and CA19–9 levels showed significant associations with OS after surgery, aligning with previous research. However, no significant correlations were found between postoperative OS and factors such as gender, age, BMI, tumour size, and tumour site. This unexpected result may stem from the unique characteristics of advanced pancreatic cancer patients within the study cohort. Many patients with resectable primary tumours had already developed distant metastases at an early stage, indicating more aggressive biological behaviour. This phenomenon highlights the distinct disease progression of advanced pancreatic cancer, likely involving different molecular mechanisms and metastatic patterns compared to early-stage disease. It suggests that advanced pancreatic cancer may harbour inherent molecular features that facilitate early metastasis, overshadowing the influence of conventional prognostic factors. Future studies should focus on elucidating these molecular mechanisms, including specific gene mutations, abnormal methylation patterns, and activated signalling pathways, to inform the development of targeted therapeutic strategies.

In addition to their prognostic value, CA19–9 and CEA demonstrated good diagnostic performance for advanced pancreatic cancer, with AUC values of 0.886 and 0.858, respectively. This supports their potential utility in clinical screening and early diagnosis within high-risk populations.

Despite these significant findings, several limitations should be acknowledged. As a single-centre retrospective study with a relatively small sample size, generalizability may be limited, and the use of an independent validation cohort was precluded. The single-centre design might also introduce selection bias. Additionally, retrospective data collection carries the inherent risks of incomplete follow-up and missing laboratory information. It is recognized that diabetes mellitus may influence CA19-9 levels independent of malignancy. Previous studies have reported that CA19-9 cut-off values for detecting pancreatic cancer may be higher in patients with diabetes, particularly in those with poor glycaemic control, compared to non-diabetic individuals (36, 37). However, diabetes status was not consistently recorded in our dataset, preventing a subgroup analysis. We cannot exclude the possibility that glycaemic status affected CA19-9 levels in some patients; nevertheless, the cut-off value we identified (37.44 U/mL) is consistent with previously reported thresholds in non-diabetic populations, suggesting its prognostic value is not driven solely by glycaemic status. Future prospective studies should include detailed diabetes history and glycaemic control data to further clarify this relationship (38). Lewis genotyping was not performed in this study, so the potential inclusion of such patients cannot be ruled out. Nevertheless, CA19–9 was utilized for prognostic stratification rather than as a standalone diagnostic test, and its prognostic value in Lewis-negative patients has been validated in larger cohorts. Future studies incorporating Lewis genotyping and diabetes history would further refine the clinical utility of CA19–9 in these subgroups. External validation in larger, multicentre prospective cohorts is essential to confirm these findings.

## 5 Conclusion

In conclusion, this study identified elevated CA19-9 level as an independent risk factor for overall survival in patients with advanced pancreatic cancer (HR = 2.059, 95% CI: 1.185–3.576, P = 0.010). Although CEA showed diagnostic potential, it did not retain independent prognostic significance after adjustment for CA19-9 (P = 0.266). The determination of optimal cut-off values for CA19-9 (37.44 U/mL) and CEA (7.16 ng/mL) provides a valuable quantitative tool for predicting patient prognosis and guiding clinical decision-making.

## Data Availability

The original contributions presented in the study are included in the article/supplementary material. Further inquiries can be directed to the corresponding author.
